# Blood meal sources and bacterial microbiome diversity in wild-caught tsetse flies

**DOI:** 10.1038/s41598-020-61817-2

**Published:** 2020-03-19

**Authors:** Alex Gaithuma, Junya Yamagishi, Kyoko Hayashida, Naoko Kawai, Boniface Namangala, Chihiro Sugimoto

**Affiliations:** 1 Division of Collaboration and Education, Research Center for Zoonosis Control, Hokkaido University, Sapporo, Japan; 2 Department of Paraclinical Studies, School of Veterinary Medicine, University of Zambia, Lusaka, Zambia

**Keywords:** Biodiversity, Microbial ecology, Microbiome, Symbiosis

## Abstract

Tsetse flies are the vectors of African trypanosomiasis affecting 36 sub-Saharan countries. Both wild and domestic animals play a crucial role in maintaining the disease-causing parasites (trypanosomes). Thus, the identification of animal reservoirs of trypanosomes is vital for the effective control of African trypanosomiasis. Additionally, the biotic and abiotic factors that drive gut microbiome diversity in tsetse flies are primarily unresolved, especially under natural, field conditions. In this study, we present a comprehensive DNA metabarcoding approach for individual tsetse fly analysis in the identification of mammalian blood meal sources and fly bacterial microbiome composition. We analyzed samples from two endemic foci, Kafue, Zambia collected in June 2017, and Hurungwe, Zimbabwe sampled in April 2014 (pilot study) and detected DNA of various mammals including humans, wild animals, domestic animals and small mammals (rat and bat). The bacterial diversity was relatively similar in flies with different mammalian species DNA, trypanosome infected and uninfected flies, and female and male flies. This study is the first report on bat DNA detection in wild tsetse flies. This study reveals that small mammals such as bats and rats are among the opportunistic blood meal sources for tsetse flies in the wild, and the implication on tsetse biology and ecology needs to be studied.

## Introduction

Tsetse flies (*Glossina spp*.) are the vectors that transmit unicellular protozoa of genus *Trypanosoma* that cause human African trypanosomiasis (HAT), also known as sleeping sickness and animal African trypanosomiasis (AAT, also known as *Nagana*). They are obligate blood-feeders that serve as a ‘transporters’ and provide an environment where the parasite can differentiate, multiply, and become infective to mammals. Some studies have revealed that the ingestion of trypanosomes during a blood meal does not always result in mature infections^1–3^. Preference for tsetse fly mammalian hosts (human, wild, or domestic animals) can differ greatly depending on the species of *Glossina*, wildlife, and geographical locations^4–6^. Tsetse flies may originally have been reptile feeders lining in forests, later becoming adapted to feeding on mammals. Earlier reports indicate that warthogs facilitated tsetse flies to migrate from the ancient forest habitat (possible origin) to the Savannah habitat^7^. The *morsitans* subgenus is found in woodland Savannah and bushy areas where there is plenty of game animals. A review of the natural hosts of 17 tsetse species and subspecies from different regions of Africa collated from published and unpublished work from 1953 to 1991, comprising altogether 47, 697 blood meals revealed that *Glossina morsitans* mainly prefer warthog as a blood meal source and bovids were found to be important especially Kudu, buffalo, bushbuck, and elands^8^. The *morsitans* subgenus can change behavior and live off domestic animals, especially cattle, in the absence of game animals. This behavior has been reported in Sudan (Koalib Hills pocket, now exterminated, where practically the only game is baboons, monkeys, and hyrax); and in Nigeria, where these tsetse flies used to live along a busy cattle trekking route passing through a densely populated and heavily cultivated area, well outside its usual range^9^. It has also been observed that *G. morsitans* do not readily change its habitats to feed on man and his domestic animals and is one of the species that disappear when the human population in an area grows^10^. On the other hand, the *palpalis* subgenus, usually residing in riverine vegetation, are closely associated ecologically with reptiles but opportunistically feed on mammals in their habitats including humans^10^. Given the difference in blood composition between various mammalian hosts, blood meals ingested from different host species could affect the structure of gut microbiota in tsetse flies, which could explain some of the previously observed geographical variations^11^. Blood source and composition may, therefore, be essential factors that modulate vector competence, through complex interactions between nutrients, immunity and bacterial communities. An earlier study found that delipidation of serum has a similar effect as complete removal of dietary serum from pig and cattle blood and significantly reduced the maturation rate of *T. congolense* infections in *G. morsitans*^12^. Another study found that blood from different sources resulted in differences in infection rates in *G. m. centralis* and *G. m. morsitans* indicating that the presence of species-specific factors in the blood affects trypanosome survival in tsetse^13^.

Blood meal collection and identification is essential for determining hosts of tsetse flies for epidemiological studies and control. Earlier studies used serological techniques for the identification of host. However, DNA-based methods are preferred due to their specificity and accuracy in the identification of vertebrate species. Mitochondrial genes have been reliable and popular targets for identifying the vertebrate species because they are maternally inherited organelles, each contains independent genomes and are numerous per cell. Two common mitochondrial gene markers, Cytochrome b (cytb) and cytochrome c oxidase I (COI), are widely used for blood meal identification of mosquitoes, black flies, tsetse flies and fleas^14^. The 12 S gene is a small subunit of ribosomal DNA in mitochondrial DNA and is one of the conserved genes between taxa, making it helpful for phylogenetic analysis^15,16^.

Additionally, this gene has an evolutionary rate that is almost similar to that of mitochondrial DNA itself^17^. Gel analysis can give a broad classification of species by the size of the amplicons but is not as specific as sequencing the amplicon. Recently with the introduction of high-throughput sequencing, multiple amplicons from multiple environmental samples can be analyzed in a single sequencing run, making identification of vertebrate species in the environment^18,19^. This metabarcoding approach is an efficient and sensitive tool for biodiversity monitoring and can be expanded to the identification of blood meal sources since it is less arduous and has a high-cost performance.

Tsetse flies harbor a natural ability to clear trypanosome infections (refractory phenotype), resulting in only few flies (<5%) that successfully transmit trypanosomes^20^. One important factor contributing to this refractory phenotype is the endogenous bacterial microbiome, which plays a role in the structural integrity of the peritrophic matrix (a lining of the fly midgut that serves as a physical barrier between the luminal contents from immune responsive epithelial cells) during larval development^21^. The colonization of the gut by microbial communities may or may not increase tsetse fly resistance against trypanosome invasion. Previous work suggests that *Sodalis* and *Wigglesworthia* (both maternally transmitted symbionts of tsetse flies) can modulate trypanosome development^22^. *Wigglesworthia* must be present in the larval stage during the maturity of tsetse flies for their immune system to properly develop and function^23^. The artificial elimination of *Wigglesworthia* results in flies being sterile and a compromised immune system leading to increased susceptibility to trypanosome infection^24^. A recent study found that disruption of *Wigglesworthia* folate production resulted in reduced sensitivity to trypanosome infection^25^.

In contrast, the presence of *Sodalis* is associated with increased susceptibility to trypanosome infection in tsetse flies and that some *Sodalis* genotypes are related to the ability of trypanosomes to establish infection in the tsetse fly midgut and that the density of these bacteria also influences susceptibility^26,27^. The presence of another tsetse fly symbiont, *Wolbachia*, is associated with cytoplasmic incompatibility (CI). A recent study demonstrated that mating *Wolbachia* infected male *G. m. morsitans* with uninfected female leads to arrested embryonic development where deformed embryos aborted without hatching into larva^28^. The presence of some bacteria can affect the appearance of others in the midgut of tsetse flies. In *G. morsitans* group, *Wolbachia* and *Spiroplasma* co-infections do not occur in nature, and *Spiroplasma* might be affected by the presence of *Wolbachia*^29^.

Moreover, the presence of some other bacterial species can hamper trypanosome infection in the midgut of tsetse flies. A recent study observed that *Kosaconia cowanni* Zambiae inhibits trypanosome in the midgut of *G.m. morsitans*^30^. Tsetse flies may ingest bacteria within the environment, particularly from the skin surface of hosts during blood meals^31,32^. The diversity and composition of these bacteria significantly vary with different regions, tsetse populations, and species. A recent study found that flies residing in geographically separated niches had different *Spiroplasma* infection prevalence^33^. Other bacteria found in tsetse flies are environmentally acquired, such as members of the phyla *Actinobacteria Bacteroidetes*, *Firmicutes*, and *Proteobacteria* are found consistently in different tsetse species captured in geographically distinct localities^34–36^. In most cases, however, these microbes account for less than 1% of tsetse’s entire enteric microbiota^34^, and their effect on the biology of tsetse flies is still unclear.

In a previous study, we have analyzed the trypanosome species in tsetse flies caught in Zambia and Zimbabwe using the DNA metabarcoding method. In this study, we applied a similar approach for the simultaneous identification of mammalian blood meal sources and bacterial microbiome in the same group of samples. The method makes use of multiple PCR of three target regions, (1) 16 S rRNA genes for bacterial analysis and (2) mitochondrial 12 S rRNA genes for mammalian host analysis coupled to high-throughput amplicon sequencing. Such an approach could be applied to study the different epidemiological variables with reduced cost and without compromising on the sensitivity and specificity of species identification. Such an approach could also help to provide more data and information essential for the formulation of new or better strategies for controlling the transmission of African trypanosomes.

## Materials and methods

### Sample collection and extraction of DNA

**Figure 1:**
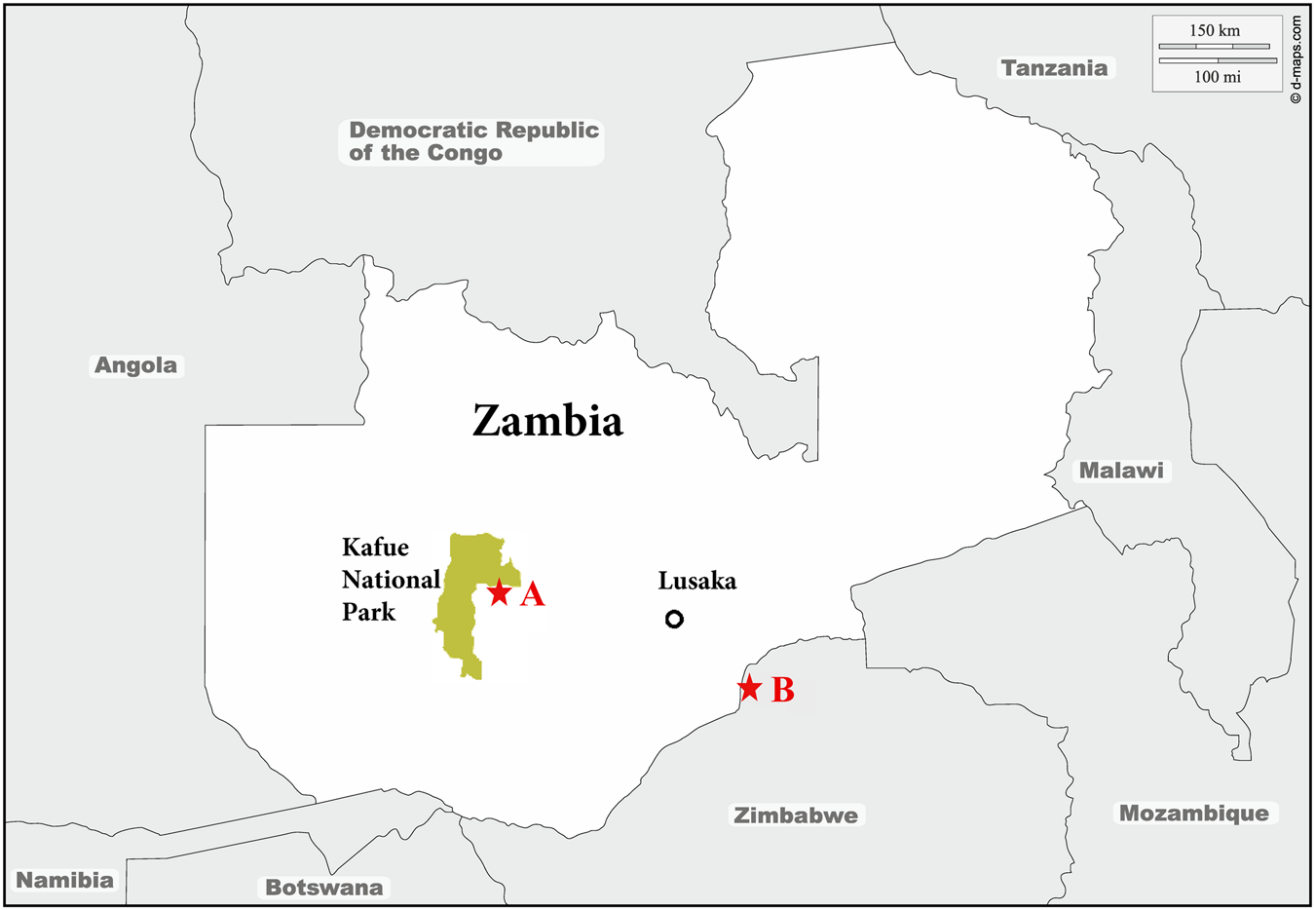
Map showing areas of tsetse fly collection marked in red stars: (**A**) Kafue in Zambia, and (**B**) Hurungwe in Zimbabwe. The map was sourced from (d-maps) https://d-maps.com/m/africa/zambie/zambie07.pdf and modified in Adobe Illustrator v23.0.1.

We analyzed 85 tsetse flies collected from Zambia, along the Kafue national park border in June 2017 (Fig. 1) hand-caught (gloved hand) in a slow-moving vehicle. The species of all these flies were identified morphologically as *Glossina morsitans centralis*. As a pilot study, we had analyzed 32 tsetse flies collected using Epsilon traps in April 2014 from Hurungwe Game reserve (near Mana pools) in Zimbabwe in order to evaluate the next-generation sequencing approach for blood meal host identification. This group of flies were analyzed as a pilot study. For this group of samples, information on tsetse fly species and sex was not available. All flies had been preserved in individual tubes containing silica gel beads before being crushed. Isolation of DNA was performed using the DNA Isolation Kit for mammalian blood (Roche USA) as per the manufacturer’s protocol for extraction of DNA from Buffy coat. However, the red blood cell lysis step was bypassed to allow lysis of all cells in the solution at once, including trypanosomes. The DNA samples were stored at −80 °C until analysis.

### Paired-end library preparation

For the analysis of mammalian host species and bacterial microbiome, a two-step PCR protocol for sequencing library preparation was applied to all analyses. The first step involved specific amplification of mammalian 12 S mitochondrial rRNA and bacterial 16 S rRNA target genes, and the second step involved Illumina-based indexing PCR allowing the samples to be pooled together for sequencing. Primers used in this study are listed in Table 1. For blood meal source analysis, we utilized primers specific for the mitochondrial 12 S ribosomal RNA gene to detect mammalian hosts. We used the 16 S ribosomal RNA V3–V4 region primers for bacterial microbiome analysis.

**Table 1:** Primers used in this study.

Primer’s target region	Primer name	Primer sequence (5′-3′)	Reference
Mitochondrial 12 S ribosomal RNA gene	MiMammal‐U forward	GGGTTGGTAAATTTCGTGCCAGC	^19^
	MiMammal‐U reverse	CATAGTGGGGTATCTAATCCCAGTTTG	
Bacterial V3 and V4 region of 16 S ribosomal RNA genes	16 S Amplicon PCR forward Primer	CCTACGGGNGGCWGCAG	^37^
	16 S Amplicon PCR reverse Primer	GACTACHVGGGTATCTAATCC	
Internal transcribed spacer 1 of Trypanosome ribosomal RNA genes	AITSF forward Primer	CGGAAGTTCACCGATATTGC	^38^
	AITSF forward Primer	AGGAAGCCAAGTCATCCATC	
Illumina Multiplexing adapter for PE library	Forward primer adapter	ACACTCTTTCCCTACACGACGCTCTTCCGATCTNN[**Forward primer**]	
	Reverse primer adapter	GTGACTGGAGTTCAGACGTGTGCTCTTCCGATCTNN[**Reverse primer**]	

^a^[·] indicate where the respective primer is attached to the respective multiplexing adapter.

Initial PCR reactions were carried out with adapter-ligated primers, where respective multiplexing adapter sequences were added to the 5′ end of each respective primer set (see Table 1). Sequencing libraries were prepared according to the Illumina metagenomic library preparation guide^37^ with slight modifications. The first PCR was done in 20 µL primary reactions that contained 0.5 µL of 10 µM of each of the forward and reverse primers, 10 µL of 2X Ampdirect Plus buffer (Shimadzu, Kyoto, Japan), 0.16 µL of 5 U/μL KAPA Taq polymerase (Kapa Biosystems, Boston, USA), 0.4 µL of DMSO, and 1 µL of template DNA. The temperature and cycling profile included incubation at 95 °C for 10 min followed by 37 cycles as follows: 95 °C for 30 sec, annealing for 1 min at 60 °C for 12 S mitochondrial RNA PCR (for blood meal host analysis) and 55 °C for bacterial 16 S RNA PCR (for bacterial microbiome analysis), extension at 72 °C for 2 min, and a final extension at 72 °C for 10 min. After the first PCR, Agencourt AMPure XP beads (Beckman Coulter, USA) were used to purify the amplicon away from free primers, primer dimer species, and PCR reagents. PCR, amplicons were viewed on 2% agarose gels. A negative template control was included in each set of PCR reactions.

The second PCR was done in 10 µL reactions containing 1 µL of 10 µM Illumina dual-index primer mix (i5 and i7 primers), 1.2 µL of 25 mM MgCl_2_, 0.4 µL of 10 mM of dNTP mix, 0.1 µL of 5 U/μL KAPA Taq polymerase (Kapa Biosystems, Boston, USA), 4 µL of 5X buffer, and 2 µL of template (amplicon from first PCR). The temperature and cycling profile included incubation at 95 °C for 3 min, followed by 11 cycles as follows: 95 °C for 30 sec, 61 °C for 1 min, 72 °C for 2 min, final extension at 72 °C for 10 min. To enable the sequencing of all amplicons in this study in one run, we used unique sets of dual index primers for each sample for each PCR analysis (blood meal and bacterial 16 S). A PCR clean-up was done using Agencourt AMPure XP beads and products viewed on 2% agarose gels.

### Amplicon library sequencing

The processing of the amplicon library for sequencing was prepared as outlined in a previous study^38^. Equal volumes of each sample amplicons from the two PCR analyses were pooled into one library. The library pool was purified using the Wizard SV Gel and PCR Clean-Up System (Promega, Madison, WI, USA) by cutting out bands of interest to separate them from primer dimers and post PCR reagents. Quantification of each of the libraries was done using a Qubit dsDNA HS assay kit and a Qubit fluorometer (ThermoFisher Scientific, Waltham, MA, USA). The concentration of the library was then adjusted to a final concentration of 4 nM using nuclease-free water and applied to the MiSeq platform (Illumina, San Diego, CA, USA). Sequencing was performed using a MiSeq Reagent Kit v3 for 300 base pairs, paired-end (Illumina, San Diego, CA, USA) and a PhiX DNA spike-in control added to improve the data quality of low diversity samples, such as single PCR amplicons. For trypanosome species detection assay, positive template controls comprising an artificial mixture of *T. congolense* and *T. brucei rhodesiense* DNA from cultured parasites were also included in the sequencing library. The Raw data of this study is available in the Sequence read archive (SRA) database under submission numbers SUB6078288 and SUB6078557.

### Sequence analysis

Analysis for the identification of trypanosome species in all the samples had been conducted in an earlier study using a new approach utilizing internal transcribed spacer 1 (ITS1) PCR and next-generation amplicon sequencing^38^. For mammalian species analysis, pre-processing of reads was done using the amplicon toolkit (AMPtk, version 1.4.0) pipeline^39^. The pre-processing involved trimming of primers, removal of sequences less than 200 bp, and merging pair-end reads. Reads were merged using the VSEARCH option for merging a long overlap region between the paired reads (~270 bp), allowing for merging of staggered read pairs. When reads did not merge, the forward read was rescued for downstream processing since the full amplicon insert could be covered by one read-only. For bacterial microbiome analysis, read merging was done using the AMPtk pipeline’s USEARCH merging option with the customized parameters: *fastq_pctid* set to 80, (minimum %id of alignment), *minhsp* set to 8, and *fastq_maxdiffs* set 10 to limit the number of mismatches in the alignment to 10, and *fastq_pctid* set to 300. For bacterial microbiome analysis, only merged reads were considered for downstream processing.

All merged reads were processed using DADA2 v1.12 in the R environment, including filtering low quality reads, removal of reads shorter than 100 bases pairs, read error correction, clustering and chimera removal resulting in Amplicon sequence variant (ASV) table representing all unique sequence clusters and their frequency per sample. About 42% of the samples from Kafue and 50% of the samples from Hurungwe had over half of their reads passing pre-processing analysis. For bacterial microbiome analysis, 77% of the samples had 90% of their reads passing pre-processing analysis. (Supplementary Table S1).

The taxonomy assignment of ASVs from the mammalian host analysis was done by BLAST^40^. The reliability of the species assignments was evaluated based on the ratio of total alignment length and percent identity to the top hit sequence. For easier analysis of data, ASVs of the same species were collated together. Taxonomy assignment of ASVs from the bacterial analysis was done using the EzBioCloud’s 16 S database https://www.ezbiocloud.net/resources using QIIME 1.9’s *parallel_assign_taxonomy_uclust.py* command.

### Data analysis and statistics

Downstream analysis and visualization of plots were done in the R environment using the phyloseq package. Read counts were normalized across samples using the DESeq2 package. Chao1 richness estimates (abundance-based estimator of the number of species in a community or species richness) and Shannon diversity values (estimator of species richness and species evenness in a community with more weight on species richness) were computed with the significance between groups tested using the non-parametric Kruskal–Wallis rank test. The variation in the taxonomic composition and abundance of bacterial species in different groups of flies (male vs. female, trypanosome infected vs. uninfected and single, multiple, or no mammalian host identified) was estimated by beta diversity analysis using principal coordinates analysis (PCoA) with the estimation done using Bray-Curtis dissimilarity distance matrix to estimate the compositional dissimilarity between two different groups or sites, based on counts. The homogeneity of group variances was first done using the *betadisper* function in vegan (R package). The significance of taxonomic composition and abundance of bacterial species between homogeneous groups was then tested using PERMANOVA. The differentially abundant taxa among the different groups of flies were estimated using DESeq2 (v.1.14.1)^41^ by performing the Wald significance test with a parametric fit type and multiple-inference correction by B-H method. Abundance plots for all bacterial genera (DESeq2 transformed counts) were plotted in the R environment. The final ASV tables and taxonomic files for mammalian host species analysis and 16 S bacterial analysis are provided (Supplementary Tables S2, S3 and S4)

## Results

### Bats, rats, and other mammals are sources of blood meal for wild tsetse flies

**Figure 2:**
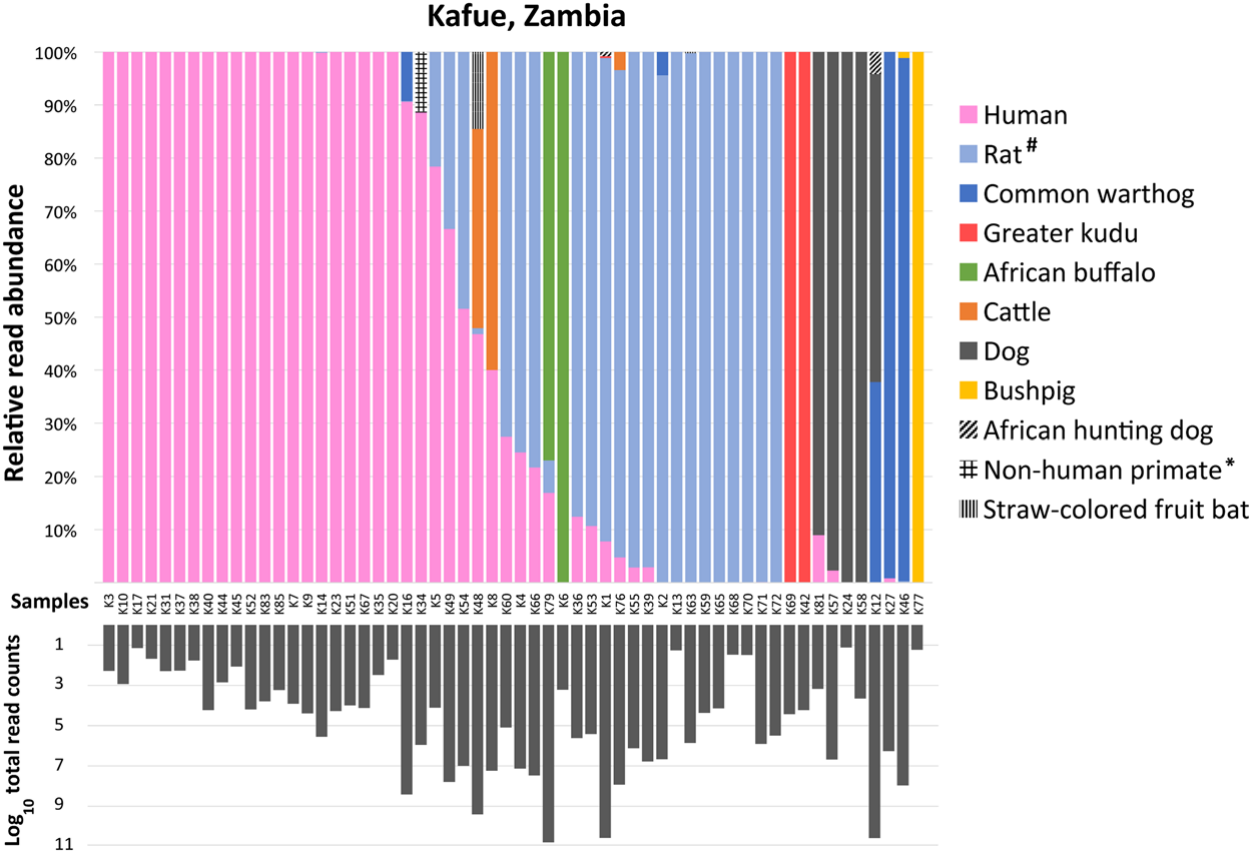
Detection of mammalian DNA in individual wild-caught tsetse flies. Flies caught in Kafue, Zambia (*n* = 85). The top graph shows the proportion of the reads for each mammalian species detected in a sample. The bottom chart shows the Log_10_ of the total read counts per sample. ^*^The best hit blast sequence had a low percent identity (91%) to a sequence from Rhesus monkey; thus, it is most likely from a non-human primate. ^#^includes sequences that had a match to *Rattus rattus* (>95% identity) and *Bandicota indica* (<95% identity).

A variety of mammalian species comprising both domestic and wild animals were detected in the tsetse fly samples. Tsetse flies caught in Kafue, Zambia, (all *G. m. centralis*) tested positive for human, cattle, dog, bush pig, African buffalo, warthog, greater kudu, rat, and bat (Fig. 2) and majority of the flies were trypanosome positive (Table 2). Flies analyzed in the pilot study (flies caught in Hurungwe, Zimbabwe) tested positive for waterbuck, bush pig, warthog and greater kudu (Supplementary Fig. S1A). This study is the first report of the detection of bat and rat sequences from wild tsetse flies. Read processing of all samples produced a final 80 ASVs. The percentage identity of 70 ASVs to their top hit sequences was above 95%, and all sequences had a query coverage of greater than 95% (Supplementary Table S5). Ten ASVs had a percent identity of below 94% to the best hit subject sequence and were considered to be from a mammalian species close to the subject sequence species.

**Table 2:** Blood meal sources and trypanosome species detected in 85 tsetse flies collected in Kafue Zambia (*G. m. centralis*).

Host DNA species detected	Kafue, Zambia (n = 85)		
	No. of flies (%)	Trypanosome positive flies	Trypanosome sp. detected in flies
**African buffalo** *(Syncerus caffer)*	2 (2%)	1	*Tg*
**African hunting dog** *(Lycaon pictus)*	2 (2%)	2	*Ts*
**Black rat** *(Rattus rattus)*	26 (31%)	9	*Tg, Ts, Tc*
**Bush pig** *(Potamochoerus larvatus)*	2 (2%)	2	*Tg, Ts, Tc*
**Cattle** *(Bos taurus)*	3 (4%)	3	*Tg, Ts*
**Common warthog** *(Phacochoerus africanus)*	5 (6%)	3	*Tg, Ts, Tc*
**Dog** *(Canis lupus)*	5 (6%)	2	*Tg*
**Greater kudu** *(Tragelaphus strepsiceros)*	3 (4%)	1	*Ts*
**Human** *(Homo sapiens)*	43 (51%)	14	*Tg, Ts, Tc, Tv*
**Straw-colored fruit bat** *(Eidolon helvum)*	2 (2%)	0	—

*Detection of human and mouse DNA in these samples could have been due to contamination, *Tb* = *Trypanosoma brucei*, *Tc* = *Trypanosoma congolense*, *Ts* = *Trypanosoma simiae*, *Tg* = *Trypanosoma godfreyi*, and *Tv* = *Trypanosoma vivax*.

Many of the flies (31 out of 32 flies) caught in Hurungwe, Zimbabwe had the mouse and human sequences detected (Supplementary Fig. S1B). The high detection rate of mouse and human DNA from the pilot study (Zimbabwe samples) was suspicious and likely from contamination with extraneous mouse DNA during sample handling (human DNA) or from laboratory contamination via equipment and reagents used (from experimental mice DNA) since they had been collected earlier for another study. Since we could not be sure of the source of human and mouse DNA in these samples, their reads were removed from further analysis, and subsequently, bacterial microbiome analysis for these samples was not done. However, wild animal DNA detected in these samples was considered to originate from blood meal since contamination with wild animal DNA was unlikely during sample processing. Extra precautions were applied during the collection of flies in Kafue, to avoid contamination of DNA from external sources. The precautions included wearing clean gloves, use of sanitized tweezers while handling the flies. The flies were placed in individual tubes containing silica gel before processing each fly individually to avoid cross-contamination. We ensured flies were processed within six days after being caught.

### **Bacterial diversity and symbiont prevalence in wild-caught*Glossina morsitans centralis***

**Figure 3:**
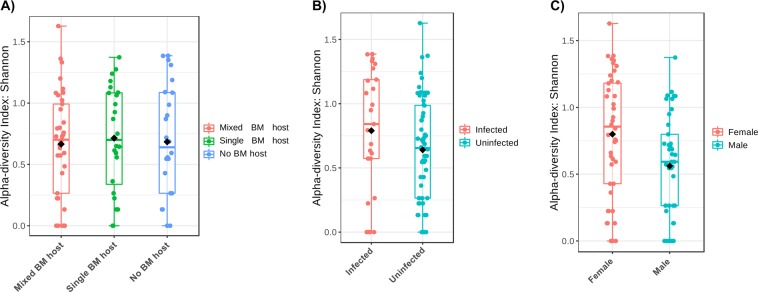
Box plots showing the alpha diversity (Shannon’s diversity) of the bacterial microbiome in wild-caught tsetse flies. The Alpha diversity values are plotted for groups of flies based on (**A**) blood meal (BM) host species detected (single (*n* = 22), mixed (*n* = 3) or no BM host (*n* = 60)), (**B**) trypanosome infection (infected (*n* = 25) or uninfected (*n* = 60)) and (**C**) sex (male (*n* = 41) or female flies (*n* = 44)).

**Figure 4:**
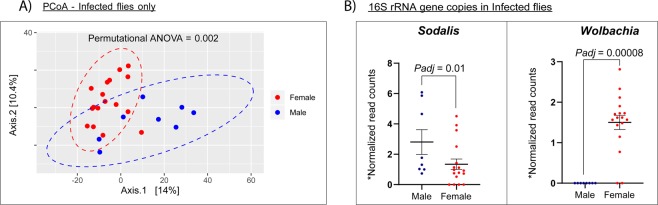
Beta diversity analysis of the bacterial microbiome and differential abundance of *Sodalis* and *Wolbachia* in trypanosome infected wild-caught tsetse flies. Charts showing (**A**) The Principal coordinates analysis (PCoA) estimation of dissimilarity in microbial flora structure in trypanosome infected male (*n* = 8) and female flies (*n* = 17). (**B**) The differential abundance of *Sodalis* and *Wolbachia* 16 S rRNA gene copies in trypanosome infected male and female flies. *Padj* = adjusted *P*-value. *The vertical axis represents Log_10_ values of (normalized read counts +1); normalization done by DSeq2 median of ratios.

**Figure 5:**
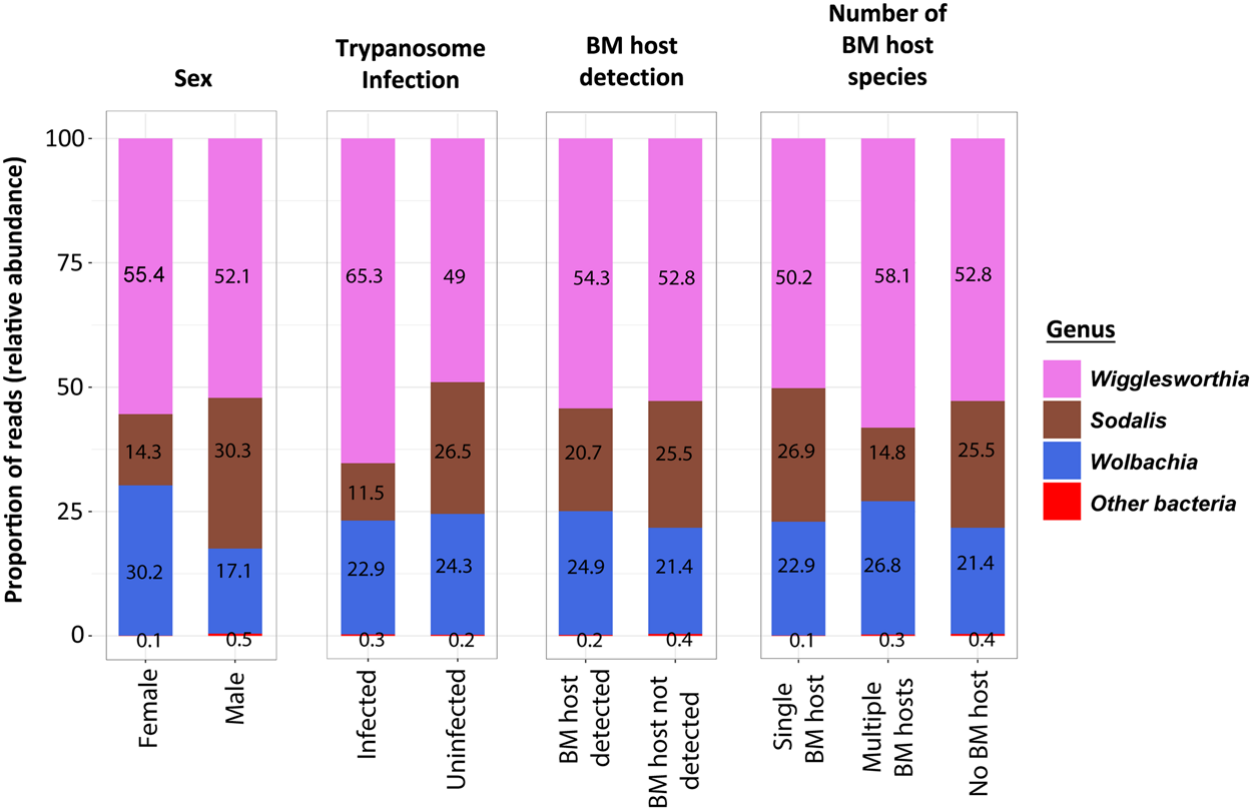
The bacterial composition (genus level) of wild caught tsetse flies from Kafue, Zambia. The proportion of reads (relative abundance) of symbiotic bacteria (*Wigglesworthia*, *Wolbachia*, and *Sodalis*) and non-symbiotic bacteria (other bacteria) for each group of flies.

Analysis of the bacterial microbiome in wild-caught *G. m centralis*, (all tsetse flies caught in Kafue, n = 85) pointed to a limited bacterial diversity of species between the different groups of flies as indicated by the Shannon diversity index (Fig. 3) and also the abundances or population of the bacterial species between the groups were not significantly different among the groups as indicated by the Chao1 estimates for species composition diversity (Supplementary Fig. S2). The bacterial composition of different groups was not significantly different, and no particular bacteria or group of bacteria was unique for any specific group as shown by beta diversity analysis estimated by principal coordinates analysis (PCoA) (Supplementary Fig. S3). However, further analysis of the distance matrix to assess the sex status and on the variance between microbial communities in trypanosome infected flies revealed a significant difference between the male and female flies (Fig. 4A). This difference was examined by differential abundance analysis with DESeq2 which showed that the difference was contributed by (a) the high abundance of *Sodalis* 16 S rRNA gene copies in infected male flies compared to females and (b) the high abundance of *Wolbachia* 16 S rRNA gene copies in infected female flies compared to males (Fig. 4B). The bacterial microbiome of the tsetse flies was dominated by the three bacteria (*Wigglesworthia, Wolbachia and Sodalis*) (Fig. 5). The prevalence of *Sodalis* was 57.7% (CI 47.0–67.6%), that of *Wolbachia* was 95.3% (CI 88.5–98.2%), and that of *Wigglesworthia* was 100% (CI 95.6–100%).

Other bacteria detected included *Bacillus*, *Pseudomonas*, *Corynebacterium, Paracoccus*, and *Acinetobacter*, among others. (Supplementary Table S6). *Yersinia*, although highly abundant, was detected in only one sample. Due to the high abundance of symbiotic bacteria (99.74% of the total reads), it was difficult to analyze the diversity of other bacteria families, which had very low read counts.

## Discussion

Our results give insight into the broad range of mammalian hosts from which tsetse flies obtain their blood meal. The flies caught in this study were collected in areas that interface human settlements and areas set aside for wild animals. Specifically, in the Kafue area, samples were collected on a road traveled by residents. This could explain the high numbers of flies with human blood meals, further supporting the submission that tsetse flies can change feeding preferences in the absence of wild animals or the presence of an alternative host^10,42^. Our results on the wildlife animals identified in this study, i.e., warthog, bush pig, African buffalo, African hunting dog, and greater kudu is in agreement with previous studies including observations made in Luangwa Valley, Zambia that reported similar mammalian hosts in *G. morsitans*^42–44^. This indicates that wild animals (mainly found in woodland Savannah and bushy areas) constitute a significant source of blood meal for tsetse flies found in Kafue, Zambia, and Hurungwe, Zimbabwe. However, the samples in this study were sampled at only one time-point in the year for each site (June 2017 for the Kafue samples and April 2014 for the Hurungwe samples). Since the presence of different mammalian hosts (especially wild animals) is variable during different seasons of the year, this may influence the number and type of mammalian blood meal host species detected in tsetse flies and their trypanosome infection rates. Due to the nature of opportunistic feeding habits, the blood meal host species can differ even in the same locality where different animals are distributed unevenly either due to natural causes or some human activities, e.g., livestock grazing and wildlife restriction.

Recently, a human case of trypanosomiasis was reported in the Kafue area (an old trypanosomiasis focus), indicating that *Trypanosoma brucei rhodesiense* is still present in the area^45^. A study in Zambia’s eastern region reported infection in dogs with *T. congolense*, *T. b. brucei* and *T. b. rhodesiense* suggesting that dogs are reservoirs of trypanosomes in Zambia^46^. More study is needed to determine the variation of mammalian hosts and specific trypanosome species prevalence to identify distinct reservoirs important for different regions. The detection of rat DNA is essential because rats are not thought to be relevant blood meal sources for the tsetse fly. The finding that rat and bat are blood meal sources of *G. morsitans* reinforces the observation that tsetse flies are opportunistic feeders. Although this is the first report of detection of DNA from fruit bat in wild-caught tsetse flies, an experimental study in 1941 had reported that *G. morsitans* and *G. tachinoides* readily fed on captured bats (*Nycteris hispida*)^47^. The presence of human infective trypanosomes in bats has not been reported, but this could be possible since an earlier study reported that experimental infection of insect-eating bats (*Tadarida condylura*) with *T. b. rhodesiense* and *T. b. brucei* produced a much more chronic infection than in mice^48^. Our data provide evidence that bats are exposed to the risk of infection by tsetse flies in the field and more studies are needed to investigate the existence of natural trypanosome infections in bats. Our finding that rats are blood meal hosts for tsetse flies was consistent with other studies that have reported *T. brucei infection* in rats in west Africa^49,50^, pointing to a possibility that wild rats may be unlooked as reservoirs of trypanosomes.

Since tsetse flies are obligate blood-feeders, they are less likely to obtain bacteria from the diet since blood is largely considered to be an entirely sterile environment. However, tsetse flies still acquire bacteria from external sources. The results obtained from this study support this observation were the three known symbionts of tsetse flies (*Wigglesworthia*, *Wolbachia* and *Sodalis*) were the most abundant comprising 99.7% of the total reads obtained while all other bacteria identified comprised only 0.3% of the total reads. This explains the results obtained from alpha and beta diversity analysis that show limited bacteria species diversity (both in abundance and richness). In this study, we report a high prevalence of *Sodalis* (57.7%) in *G. m. centralis* from Kafue, Zambia compared to the results of a previous study conducted in Luambe National Park, Zambia, that found a lower *Sodalis* prevalence of 17.5% in *G. m. morsitans*, 1.4% prevalence in *G. pallidipes*, but a higher prevalence in *G. brevipalpis* (93.7%)^51^. Additionally, the prevalence of *Wolbachia* (95.3%) was higher than a previous study that found 68.1% in *G. tachinoides* and 58.5% in *G. m. submorsitans*^52^. As earlier stated, due to sampling at only one specific time-point of the year, we cannot rule out the effect of seasonal changes on the prevalence of bacterial symbionts. We observed that the *Sodalis* and *Wolbachia* 16 S gene copies significantly different in infected male flies and infected female flies. These abundance values cannot be interpretable as actual bacterial cell numbers, due to the nature of amplicon data and the presence of multiple copies of 16 S rRNA genes in bacteria. However, information on which representative sequences have significantly different copy-number counts between groups can provide useful information and guide the generation of hypotheses. Therefore, there is need for more studies on the infection rates of *Sodalis* and *Wolbachia* in infected *G. morsitans*. The prevalence of *Wigglesworthia glossinidius* was 100% as expected since it is an obligate symbiont necessary for the immunity and reproduction of tsetse flies. Our study had some limitations in such as the low number of trypanosome infected flies as well as low and uneven numbers in groups of flies with mixed, single, or no blood meal detected. Nonetheless, the results of this study are in agreement with the observation that symbiotic bacteria account for up to 99% of tsetse fly’s entire enteric microbiota^34^. The environmentally-acquired bacteria in this study accounted for only 0.3% of the total read counts. The taxonomic identities of some of these bacteria were in agreement with previous studies that also reported *Bacillus*, *Pseudomonas*, *Corynebacterium*, and *Acinetobacter* in wild-caught tsetse flies^34,53,54^. We also detected some that are commonly found in soil and water, indicating surface contaminating bacteria (from the environment). These bacteria, however, accounted for less than 0.1% of the total read count. Owing to the low read numbers obtained for non-symbiotic bacteria, and limited samples, we could not correlate any genus with blood meal source, sex, or status of trypanosome infection. Our results seem to indicate that blood meal sources from different hosts have little effect on the general gut microbiome composition of tsetse flies. However, the imposing abundance of the three symbiotic bacteria hampers the accurate detection and analysis of other bacteria; thus, the difficulty in identifying any significant associations. Studies on other vectors indicate changes in bacterial flora with different blood meals, as has been demonstrated recently in mosquitos (*Aedes aegypti*)^55^. Future studies should focus on more sensitive detection and analysis of non-symbiotic bacteria in tsetse flies to shed light on the relationship between bacterial microbiome, trypanosome infection, blood meal source, and other factors and their importance in aspects such as vector competence, immunity, reproduction, etc.

## Supplementary information

Supplementary information.

Supplementary information2.

## References

[1] Moloo S. K., Asonganyi T. & Jenni L. Cyclical development of Trypanosoma brucei gambiense from cattle and goats in Glossina. [Internet]. Acta Tropica. Elsevier Science Available, https://hdl.handle.net/10568/28000 (1986). 2882668

[2] Dukes P, Kaukas A, Hudson KM, Asonganyi T, Gashumba JK (1989). A new method for isolating trypanosoma brucei gambiense from sleeping sickness patients. Trans R Soc Trop Med Hyg..

[3] Maudlin I, Welbum SC (1994). Maturation of trypanosome infections in tsetse, a review. Exp Parasitol..

[4] Omolo M. O. *et al*. Prospects for developing odour baits to control Glossina fuscipes spp., the major vector of human African trypanosomiasis. *PLoS Negl Trop Dis*. 3. 10.1371/journal.pntd.0000435 (2009). 10.1371/journal.pntd.0000435 PMC2674566 19434232

[5] Farikou O (2010). Tsetse fly blood meal modification and trypanosome identification in two sleeping sickness foci in the forest of southern Cameroon. Acta Trop. Elsevier B.V..

[6] Muturi C. N *et al*. Mithöfer KM, et al. Tracking the feeding patterns of tsetse flies (glossina genus) by analysis of bloodmeals using mitochondrial cytochromes genes. *PLoS One*. 6, 10.1371/journal.pone.0017284 (2011). 10.1371/journal.pone.0017284 PMC3046180 21386971

[7] Ford, J. The geographical distribution of Glossina. In: Mulligan, HW (ed) The African Trypanosomiases. London: George Allen and Unwin; pp. 274–297 (1970).

[8] Moloo SK (1993). The distribution of Glossina species in Africa and their natural hosts. Int J Trop Insect Sci. Cambridge University Press.

[9] FAO. Training manual for tsetse control personnel; Ecology and behaviour of tsetse. FAO. 1982;Volume 2. Available: ftp://ftp.fao.org/docrep/fao/009/p5444e/p5444e00.pdf

[10] Leak S (1999). Tsetse Biology and Ecology: Their Role in The Epidemiology and Control of Trypanosomiasis..

[11] Geiger A (2011). Bacterial Diversity Associated with Populations of Glossina spp. from Cameroon and Distribution within the Campo Sleeping Sickness Focus. Microb Ecol..

[12] Maudlin I, Kabayo JP, Flood MET, Evans DA (1984). Serum factors and the maturation of Trypanosoma congolense infections in Glossina morsitans. Zeitschrift für Parasitenkd Parasitol Res..

[13] Mihok S, Olubayo RO, Darji N, Zweygarth E (1993). The influence of host blood on infection rates in Glossina morsitans sspp. infected with Trypanosoma congolense, T. brucei and T. simiae. Parasitology..

[14] Kent, R. J. Molecular methods for arthropod bloodmeal identification and applications to ecological and vector-borne disease studies. *Molecular Ecology Resources*. pp 4–18. 10.1111/j.1755-0998.2008.02469.x (2009). 10.1111/j.1755-0998.2008.02469.x 21564560

[15] Matthee CA, Davis SK (2001). Molecular insights into the evolution of the family Bovidae: A nuclear DNA perspective. Mol Biol Evol..

[16] Vun VF, Mahani MC, Lakim M, Ampeng A, Md-Zain BM (2011). Phylogenetic relationships of leaf monkeys (Presbytis; Colobinae) based on cytochrome b and 12 S rRNA genes. Genet Mol Res..

[17] Rosli MKA (2011). Optimization of PCR conditions to amplify Cyt b, COI and 12 S rRNA gene fragments of Malayan gaur (Bos gaurus hubbacki) mtDNA. Genet Mol Res..

[18] Miya, M. *et al*. MiFish, a set of universal PCR primers for metabarcoding environmental DNA from fishes: detection of more than 230 subtropical marine species. *R Soc Open Sci*. 2, 10.1098/rsos.150088 (2015). 10.1098/rsos.150088 PMC4632578 26587265

[19] Ushio M (2017). Environmental DNA enables detection of terrestrial mammals from forest pond water. Mol Ecol Resour..

[20] Aksoy S, Gibson WC, Lehane MJ (2003). Interactions between tsetse and trypanosomes with implications for the control of trypanosomiasis. Adv Parasitol..

[21] Weiss, B. L., Wang, J., Maltz, M. A., Wu, Y. & Aksoy, S. Trypanosome Infection Establishment in the Tsetse Fly Gut Is Influenced by Microbiome-Regulated Host Immune Barriers. *PLoS Pathog*. 9, 10.1371/journal.ppat.1003318 (2013). 10.1371/journal.ppat.1003318 PMC3630092 23637607

[22] Geiger A, Ponton F, Simo G (2015). Adult blood-feeding tsetse flies, trypanosomes, microbiota and the fluctuating environment in sub-Saharan Africa. ISME J. Nature Publishing Group.

[23] Weiss, B. & Aksoy, S. Microbiome influences on insect host vector competence. *Trends in Parasitology*, 10.1016/j.pt.2011.05.001 (2011). 10.1016/j.pt.2011.05.001 PMC3179784 21697014

[24] Wang J, Wu Y, Yang G, Aksoy S (2009). Interactions between mutualist Wigglesworthia and tsetse peptidoglycan recognition protein (PGRP-LB) influence trypanosome transmission. Proc Natl Acad Sci..

[25] Rio, R. V. M. *et al*. Mutualist-provisioned resources impact vector competency. *MBio*. 10, 10.1128/mBio.00018-19 (2019). 10.1128/mBio.00018-19 PMC6550517 31164458

[26] Geiger A (2007). Vector competence of Glossina palpalis gambiensis for Trypanosoma brucei s.l. and genetic diversity of the symbiont Sodalis glossinidius. Mol Biol Evol..

[27] Farikou O (2010). Tripartite interactions between tsetse flies, Sodalis glossinidius and trypanosomes-An epidemiological approach in two historical human African trypanosomiasis foci in Cameroon. Infect Genet Evol..

[28] Alam, U. *et al*. Wolbachia symbiont infections induce strong cytoplasmic incompatibility in the Tsetse fly glossina morsitans. *PLoS Pathog*. 7, 10.1371/journal.ppat.1002415 (2011). 10.1371/journal.ppat.1002415 PMC3234226 22174680

[29] Doudoumis V (2017). Challenging the Wigglesworthia, Sodalis, Wolbachia symbiosis dogma in tsetse flies: Spiroplasma is present in both laboratory and natural populations. Sci Rep. Springer US.

[30] Weiss, B. L. *et al*. Colonization of the tsetse fly midgut with commensal Kosakonia cowanii Zambiae inhibits trypanosome infection establishment. *PLoS Pathog*. 15, 10.1371/journal.ppat.1007470 (2019). 10.1371/journal.ppat.1007470 PMC6394900 30817773

[31] Simo G (2008). Tsetse fly host preference from sleeping sickness foci in Cameroon: Epidemiological implications. Infect Genet Evol..

[32] Farikou O (2010). Tsetse fly blood meal modification and trypanosome identification in two sleeping sickness foci in the forest of southern Cameroon. Acta Trop..

[33] Schneider DI (2019). Spatio-temporal distribution of Spiroplasma infections in the tsetse fly (Glossina fuscipes fuscipes) in northern Uganda. PLoS Negl Trop Dis..

[34] Aksoy E (2014). Analysis of multiple tsetse fly populations in Uganda reveals limited diversity and species-specific gut microbiota. Appl Environ Microbiol..

[35] Geiger A (2009). First isolation of Enterobacter, Enterococcus, and Acinetobacter spp. as inhabitants of the tsetse fly (Glossina palpalis palpalis) midgut. Infect Genet Evol..

[36] Lindh JM, Lehane MJ (2011). The tsetse fly Glossina fuscipes fuscipes (Diptera: Glossina) harbours a surprising diversity of bacteria other than symbionts. Antonie van Leeuwenhoek, Int J Gen. Mol Microbiol..

[37] Illumina. 16 S Metagenomic Sequencing Library Preparation. Illumina.com. 1–28. Available, http://support.illumina.com/content/dam/illumina-support/documents/documentation/chemistry_documentation/16s/16s-metagenomic-library-prep-guide-15044223-b.pdf (2013).

[38] Gaithuma AK (2019). A single test approach for accurate and sensitive detection and taxonomic characterization of Trypanosomes by comprehensive analysis of internal transcribed spacer 1 amplicons. PLoS Negl Trop Dis. Public Library of Science..

[39] Palmer JM, Jusino MA, Banik MT, Lindner DL (2018). Non-biological synthetic spike-in controls and the AMPtk software pipeline improve mycobiome data. PeerJ..

[40] Camacho C (2009). BLAST + : Architecture and applications. BMC Bioinformatics..

[41] Love, M. I., Huber, W. & Anders, S. Moderated estimation of fold change and dispersion for RNA-seq data with DESeq2. *Genome Biol*. 15. 10.1186/s13059-014-0550-8 (2014). 10.1186/s13059-014-0550-8 PMC4302049 25516281

[42] Clausen PH (1998). Host preferences of tsetse (Diptera: Glossinidae) based on bloodmeal identifications. Med Vet Entomol..

[43] Laohasinnarong D (2015). Studies of trypanosomiasis in the Luangwa valley, north-eastern Zambia. Parasites and Vectors..

[44] Auty H (2012). Trypanosome Diversity in Wildlife Species from the Serengeti and Luangwa Valley Ecosystems. PLoS Negl Trop Dis..

[45] Squarre D (2016). Human African Trypanosomiasis in the Kafue National Park, Zambia. PLoS Negl Trop Dis..

[46] Lisulo, M. *et al*. Determination of the prevalence of African trypanosome species in indigenous dogs of Mambwe district, eastern Zambia, by loop-mediated isothermal amplification. *Parasites and Vectors*. 7, 10.1186/1756-3305-7-19 (2014). 10.1186/1756-3305-7-19 PMC3895695 24411022

[47] Nash TAM (1941). Bats as a Source of Food for Glossina morsitans and G. tachinoides. Bull Entomol Res..

[48] Woo, P. T. & Hawkins, J. D. Trypanosomes and experimental trypanosomaisis in East African bats. *Acta Trop*. **32**, 57–64. https://www.ncbi.nlm.nih.gov/pubmed/239552 (1975) 239552

[49] Büscher, P. *et al*. Do Cryptic Reservoirs Threaten Gambiense-Sleeping Sickness Elimination? *Trends in Parasitology*. 197–207, 10.1016/j.pt.2017.11.008 (2018). 10.1016/j.pt.2017.11.008 PMC5840517 29396200

[50] Cordon-Obras, C *et al*. Molecular evidence of a Trypanosoma brucei gambiense sylvatic cycle in the human african trypanosomiasis foci of Equatorial Guinea. *Front Microbiol*. 6, 10.3389/fmicb.2015.00765 (2015). 10.3389/fmicb.2015.00765 PMC4513237 26257727

[51] Dennis JW (2014). Sodalis glossinidius prevalence and trypanosome presence in tsetse from Luambe National Park, Zambia. Parasites and Vectors..

[52] Kame-Ngasse, G. I. *et al*. Prevalence of symbionts and trypanosome infections in tsetse flies of two villages of the “faro and Déo” division of the Adamawa region of Cameroon 06 Biological Sciences 0604 Genetics. *BMC Microbiol*. 18, 10.1186/s12866-018-1286-5 (2018). 10.1186/s12866-018-1286-5 PMC6251084 30470177

[53] Malele I (2018). Bacterial diversity obtained by culturable approaches in the gut of Glossina pallidipes population from a non sleeping sickness focus in Tanzania: preliminary results. BMC Microbiol..

[54] Griffith BC (2018). Analysis of the gut-specific microbiome from field-captured tsetse flies, and its potential relevance to host trypanosome vector competence. BMC Microbiol. BioMed Central.

[55] Muturi, E. J., Dunlap, C., Ramirez, J. L., Rooney, A. P. & Kim, C. H. Host blood-meal source has a strong impact on gut microbiota of Aedes aegypti. *FEMS Microbiol Ecol*. 95, 10.1093/femsec/fiy213 (2019). 10.1093/femsec/fiy213 30357406

