# Supplementary material for: Blood meal sources and bacterial microbiome diversity in wild-caught tsetse flies
Source: Sci Rep. 2020 Mar 19;10:5005. doi: 10.1038/s41598-020-61817-2 (PMC7081217; doi:10.1038/s41598-020-61817-2)
Supplement: Supplementary file 1 — Supplementary information. [file 41598_2020_61817_MOESM1_ESM.pdf]

Supplementary information

Blood meal sources and bacterial microbiome diversity in wild-caught tsetse flies.

Alex Gaithuma, Junya Yamagishi, Kyoko Hayashida, Naoko Kawai, Boniface Namangala and Chihiro Sugimoto

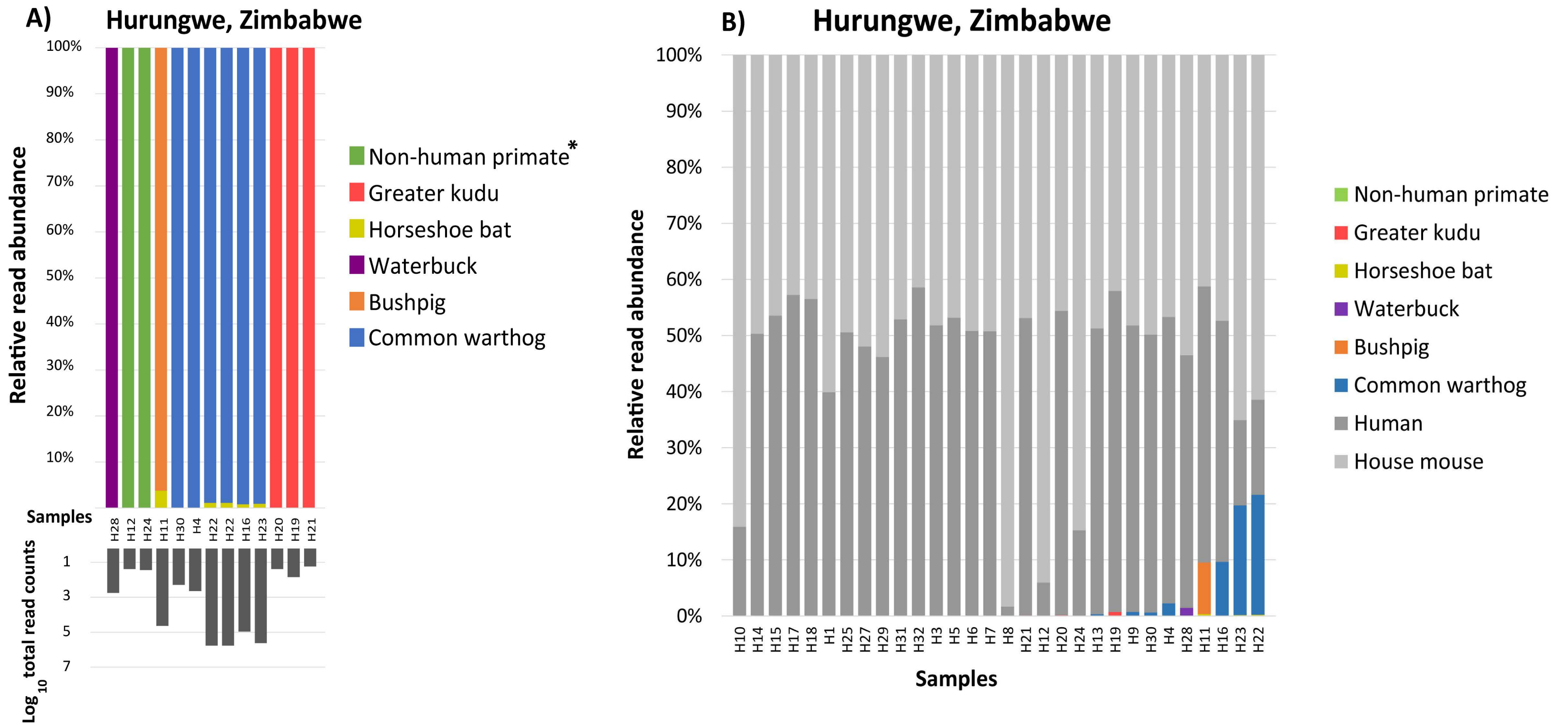

Figure S1. Mammalian species detected in flies caught in Hurungwe, Zimbabwe. (A) Bar chart showing wildlife species only without mouse and human-derived reads. (B) Bar chart showing all mammalian species detected. The gray and dark grey colors represent mouse and human-derived reads respectively detected in all samples and in high frequencies in fig (B).

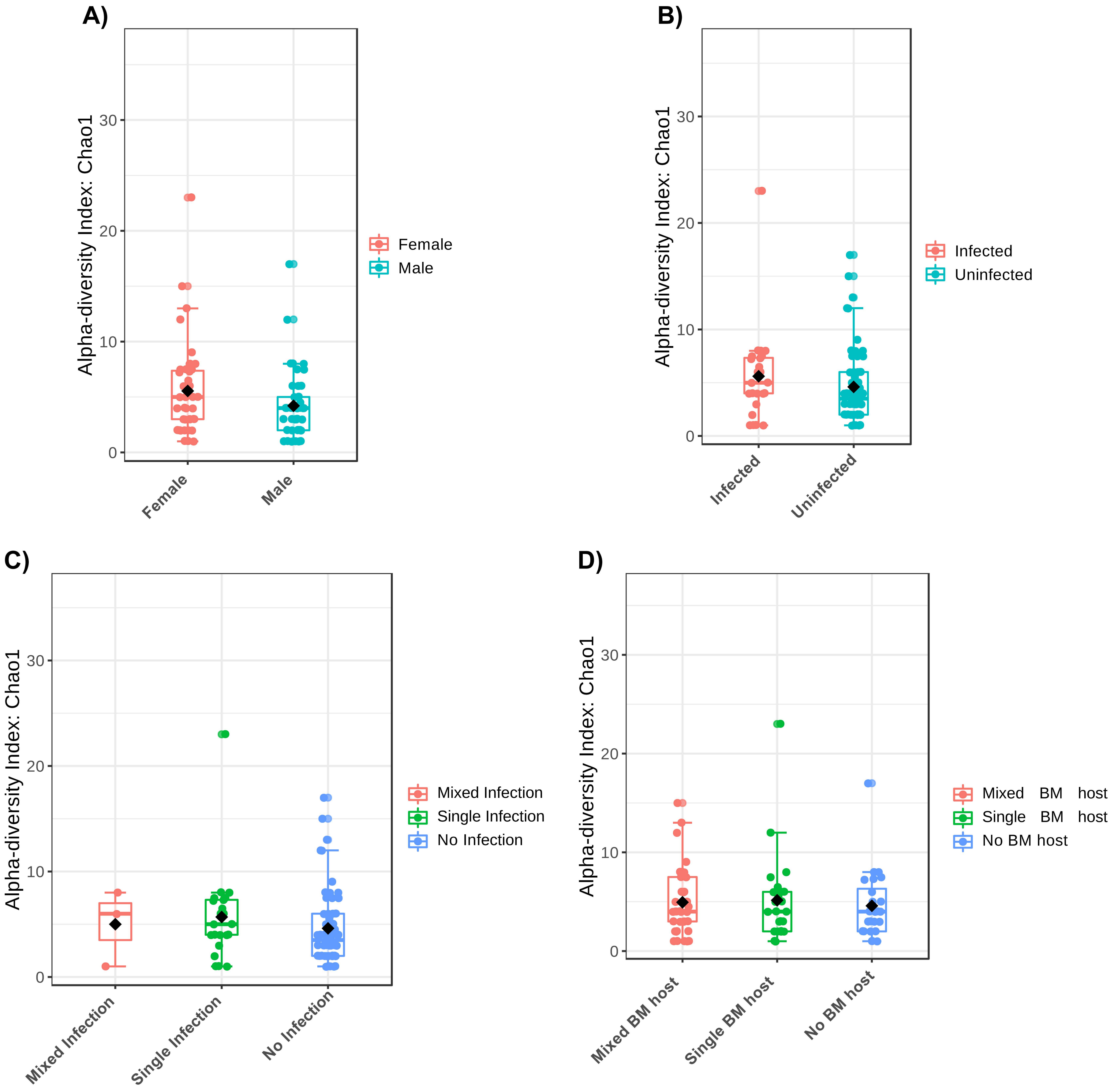

Figure S2. Alpha diversity of bacterial microbiome in wild-caught tsetse flies by Chao1 richness estimate. Plots show species richness for groups of flies based on (A) sex (male or female flies), (B) trypanosome infection (infected or uninfected), (C) trypanosome species (mixed infection, single infection or uninfected) and (D) blood meal (BM) host species detected (single, mixed or no BM host).

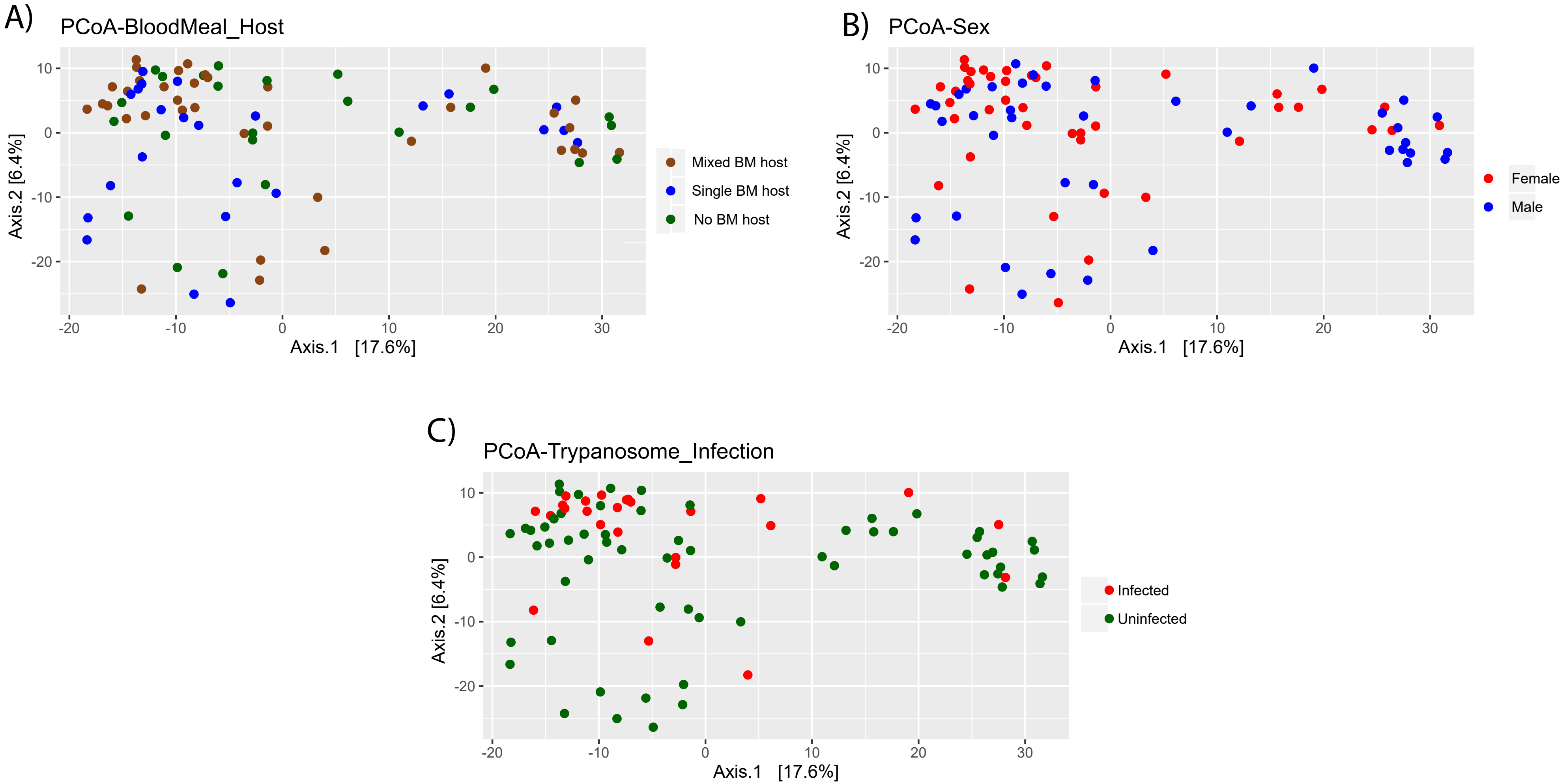

Figure S3. Beta diversity of bacterial microbiome in wild-caught tsetse flies estimated by principal coodinates analysis (PCoA). The plots show the diversity of flies grouped by (A) blood meal (BM) host species detected (single (*n*=22), mixed (*n*=3) or no BM host (*n*=60)), (B) sex (male (*n*=41) or female flies (*n*=44)) and (C) trypanosome infection (infected (*n*=25) or uninfected (*n*=60)).
